# Surgical management of stress urinary incontinence in women: safety, effectiveness and cost-utility of trans-obturator tape (TOT) versus tension-free vaginal tape (TVT) five years after a randomized surgical trial

**DOI:** 10.1186/1472-6874-11-34

**Published:** 2011-07-22

**Authors:** Sue Ross, Magali Robert, Doug Lier, Misha Eliasziw, Philip Jacobs

**Affiliations:** 1 Department of Obstetrics and Gynaecology, University of Calgary, Calgary, T2N 2T9, Canada; 2 Institute of Health Economics, Edmonton, T6G 2R3, Canada; 3 Department of Community Health Sciences, University of Calgary, Calgary, T2N 4Z6, Canada; 4 Department of Medicine, University of Alberta, Edmonton, T6G 2R3, Canada

**Keywords:** Urinary incontinence, stress/surgery, suburethral slings, female, treatment outcome, cost-effectiveness, 5 year follow-u

## Abstract

**Background:**

We recently completed a randomized clinical trial of two minimally invasive surgical procedures for stress urinary incontinence, the retropubic tension-free vaginal tape (TVT) versus the trans-obturator tape (TOT) procedure. At one year postoperatively, we were concerned to find that a significant number of women had tape that was palpable when a vaginal examination was undertaken. Because the risk factors for adverse outcomes of tape surgery are not clearly understood, we are unable to say whether palpable tapes will lead to vaginal erosions or whether they merge into vaginal tissue. We do not know whether patients go on to have further adverse consequences of surgery, leading to additional cost to patients and healthcare system. Our current study is a 5 year follow-up of the women who took part in our original trial.

**Methods/Design:**

All 199 women who participated in our original trial will be contacted and invited to take part in the follow-up study. Consenting women will attend a clinic visit where they will have a physical examination to identify vaginal erosion or other serious adverse outcomes of surgery, undertake a standardized pad test for urinary incontinence, and complete several health-related quality of life questionnaires (15D, UDI-6, IIQ-7). Analyses will compare the outcomes for women in the TOT versus TVT groups. The cost-effectiveness of TOT versus TVT over the 5 years after surgery, will be assessed with the use of disease-specific health service administrative data and an objective health outcome measure. A cost-utility analysis may also be undertaken, based on economic modeling, data from the clinical trial and inputs obtained from published literature.

**Discussion:**

This study is needed now, because TOT and TVT are among the most frequently conducted surgical procedures for stress urinary incontinence in Canada. Because stress urinary incontinence is so common, the impact of selecting an approach that causes more adverse events, or is less effective, will have a significant impact on individual quality of life, and societal and health care costs.

**Trial registration:**

ClinicalTrials.gov NCT00234754. Registered October 2005.

## Background

A 2010 Health Canada notice described "*Complications Associated with Transvaginal Implantation of Surgical Mesh for the Treatment of Stress Urinary Incontinence and Pelvic Organ Prolapse*" [1]. The warning highlighted longer term complications associated with the use of transvaginally-placed mesh for the treatment of stress urinary incontinence (SUI), including erosion of the tape through the vaginal epithelium, pain including dyspareunia, and infection. The notice also stated that risk factors associated with these complications are not completely understood. Health Canada therefore advised surgeons to seek specialist training in the use of mesh devices, to discuss possible adverse events with patients before surgery, and to warn patients that additional surgery may be required to address complications. They also noted that such surgery may not fully correct any adverse effects. A similar memo was circulated by the FDA in 2008, stating that over 1000 adverse events had been reported [2].

New devices are being introduced frequently for SUI, because it is a common condition [3-5] which is becoming more prevalent as a result of our aging population [4-6]. In addition, many patients are less tolerant of symptoms, embarrassment and inconvenience caused by SUI [6].

There has been increasing concern about the introduction of new surgical devices into urogynaecology without evidence of safety and effectiveness[7-9]. Device manufacturers are not required to provide such information to obtain a license in Canada, the USA or Europe, if a device is made by a manufacturer who makes similar products or if a similar product is already produced by another manufacturer [10-12]. Therefore it becomes the responsibility of clinicians to evaluate any known relevant evidence [7-9,13]. Unfortunately even if short-term outcome is known, longer term outcomes usually remain unknown, and therefore clinicians are unable to fully inform patients of the consequences of surgery.

Clinical trials in surgery are difficult to do, particularly when new devices are widely adopted early [13]. There is no incentive for device manufacturers to provide long-term evidence of safety and effectiveness, and it is difficult for independent researchers to obtain funding, because there is a perception that licensed devices must be safe. In addition, it is difficult to recruit patients to device trials [14,15]. Thus there is a scarcity of longer term evidence.

We recently completed a randomized clinical trial of two stress incontinence procedures, finding concerning clinical outcomes at one year, with a significant number of women having tape that was palpable when a vaginal examination was undertaken at one year [16]. Because the risk factors for adverse outcomes of tape surgery are not clearly understood [1,2], we are unable to say whether palpable tapes will lead to vaginal erosions or whether they merge into vaginal tissue. We do not know whether patients go on to have further adverse consequences of surgery, leading to additional cost to patients and healthcare system.

### The problem to be addressed

#### Stress urinary incontinence (SUI)

Urinary incontinence (UI) is a prevalent condition that affects approximately 27% of women worldwide [3,5] with far-reaching physical, psychological, social, and economic implications. Incontinence has been found to reduce health-related quality of life to roughly the same degree as chronic conditions such as depression and Type I diabetes [17]. Personal consequences include restriction of physical and social activity, self-imposed social isolation [18,19], and sexual dysfunction [20]. UI is also a major factor contributing to nursing home admission and hospital readmission among older women with co-morbid conditions [21,22]. With direct costs estimated at over 25 billion dollars per year in the United States (approximately 3,500 dollars for each incontinent person) [23,24] and 1.5 billion dollars per year in Canada [25], UI is associated with major individual, societal, and health care costs.

SUI is the most common form of UI in women 60 years of age or younger and is a contributing factor for the majority of older women with incontinence [26,27]. Women with SUI experience leakage associated with increases in intra-abdominal pressure, such as with physical exertion, coughing, laughing, and/or sneezing. Physiological factors include anatomical defects in pelvic support structures and/or neuromuscular dysfunction affecting urethral pressure [28]. Women with a family history of SUI are twice as likely to have SUI compared to those without a family history [5]. A predisposition gene for pelvic floor disorders including SUI was recently described [29]. First line treatment is usually conservative, for example for example pelvic floor muscle exercises [30]. In cases where conservative therapies fail, women may opt for surgical treatment [31]. Surgical treatments for SUI are among the most common of all female surgeries [32].

#### Tape surgeries for SUI

Since it was introduced in 1996, the minimally invasive retropubic tension-free vaginal tape (TVT) [33-41] has become the surgery of choice for treating stress urinary incontinence. Concern about complications associated with TVT [37-41] led in 2001 to the development of another minimally invasive procedure using the trans-obturator tape (TOT) procedure [42].

Prior to introducing TOT into clinical practice in Calgary, we conducted a trial of 52 TOT procedures, using the Obtape device. At 12 months postoperatively, the vaginal erosion rate was found to be unacceptably high (15%) [43]. We believed that the high rate of erosions was associated with the specific polypropylene mesh (pore size 50 μm) used in the Obtape device [44], but we remained concerned that the trans-obturator approach itself could be at fault. We decided to undertake a randomized trial comparing TOT to TVT using surgical devices from a single manufacturer, that incorporated a type of polypropylene mesh which was completely macroporous (pore size >75 μm), to permit infiltration by macrophages, fibroblasts and blood vessels [44]. Our trial was designed to compare the effectiveness of the surgical approach of TOT to TVT in terms of objective cure of stress urinary incontinence (SUI) at 12 months postoperatively (NCT002237540) [16]. We also conducted a health economic evaluation alongside the RCT [45].

In our RCT, women with SUI were randomly allocated to either TOT or TVT procedures, and reviewed at 12 months after surgery. Primary outcome was objective evidence of "cure" evaluated by standardized pad test ("cure" defined as <1 g urine leaked). Other outcomes included: complications; subjective cure; incontinence-related quality of life; return to usual sexual activity; and satisfaction with surgery. Primary analysis compared the proportion of patients in each group who were cured at 12-month follow-up.

One hundred and ninety-nine women were randomized (94 in the TOT, 105 in the TVT group). 68 women (81%) in the TOT group were cured, versus 67 (77%) in the TVT group (RR 1.05, 95%CI 0.90 to 1.23, p = 0.58). On vaginal exam, the tape was palpable for 68 women (80%) in the TOT and 24 (27%) in the TVT group (RR 0.22, 95%CI 0.13 to 0.37, p < 0.001), and more women in the TOT group experienced groin pain during vaginal palpation (13 (15%) versus 5 (6%) in the TVT group, p = 0.04). Quality of life improved significantly from baseline in both groups (30 point improvement in IIQ-7 score, both groups).

At 12 months, the majority of women had minimal leakage, and their quality of life had improved significantly, but differences were not observed between groups. The presence of palpable tape, particularly among the TOT group, was concerning: longer follow-up is needed to estimate if this outcome leads to vaginal erosion or resolves over time. We concluded that until long-term follow-up is available, TVT should remain the mid-urethral sling procedure of choice [16].

Our economic evaluation was a cost utility analysis, using data collected in the trial, as well as administrative utilization and cost data [45]. The findings from the cost utility analysis suggest that TOT is a cost-effective alternative to TVT in the treatment of SUI, perhaps as a result of underlying difference in utilization of various health services following randomization [45].

#### Synthesis

The clinical results from our RCT raise the possibility that the TOT approach may lead to more women having longer term problems if those with tapes found to be palpable at 12 months go on to develop vaginal erosion. However our economic evaluation suggested that TOT may be more cost effective than TVT. The apparently contradictory results from our RCT and economic evaluation must be further investigated, to determine whether the TOT approach is safe and effective.

### Principal research questions

#### Primary question: safety

What is the incidence of vaginal erosion or other serious adverse outcomes of surgery among women who had a surgical procedure for stress urinary incontinence utilizing a TOT device, versus a TVT device, over the 5 years following surgery?

#### Secondary question: effectiveness

What are the objective and subjective outcomes of TOT compared with TVT at 5 years following surgery for SUI?

#### Secondary questions: health economics

What are the disease-specific rates of health service utilization related to repeat surgical intervention, as well as surgery and other treatment related to adverse events for women who had a TOT procedure, versus those who had a TVT procedure, over the 5 years after surgery?

Using economic modeling and cost utility analysis, is TOT cost-effective compared with TVT over the 5 years after surgery?

#### Other question

Do women with tape found to be palpable in the vagina at 12 months postoperatively, go on to develop vaginal erosion by 5 years following surgery?

### Literature review

Unfortunately there is very little evidence from clinical trials related to follow-up of patients for longer than a year after TOT versus TVT surgery.

#### Safety

A recent Cochrane review found that minimally invasive suburethral sling procedures (such as TOT and TVT) used for the surgical management of SUI produced more bladder perforations (TVT), but fewer other operative or short-term postoperative complications than traditional surgical procedures (such as slings, retropubic colposuspension and laparoscopic colposuspension) [46]. The review stated that minimally invasive suburethral sling procedures are the management of choice for SUI.

Three published systematic reviews with meta analyses compared complications rates associated with TOT versus TVT [47-49]. The reviews overlapped, presenting information on short-term outcome: Sung's study included six RCTs [47], Novara's included 14 [48], and Latthe's included 11 [49]. The TOT procedure produced fewer bladder perforations and hematomas, but more patients had groin pain at two months postoperatively. The meta analyses did not demonstrate significant differences in erosion rates or overactive bladder. Few of the included trials reported longer term follow-up (over 12 months), and the outcomes evaluated varied widely. In addition, most of the studies were described as being of limited methodological and clinical quality, and therefore the authors recommended that high-quality studies with longer term follow-up were needed [47-49]. A further review of literature published between January 2008 and January 2009 (including six RCTs) came to similar conclusions [50].

Despite the large number of trials of TOT versus TVT, the lack of long-term follow-up prevents surgeons and patients from being able to make treatment decisions on the basis of evidence of safety.

#### Effectiveness

The recent Cochrane review found that minimally invasive suburethral sling procedures (such as TOT and TVT) are as effective as traditional surgical procedures in the short-term [46]. Sung, Novara, Latte and Long examined the evidence of short-term effectiveness of TOT versus TVT, concluding that despite the large volume of research, there is no clear evidence to suggest that one of the two procedures is preferable to the other [47,49-51]. The maximum previously reported follow-up was 36 months [51]. The four reviews concluded that longer term follow-up is needed before a recommendation can be made regarding effectiveness.

#### Health economics

Despite the acknowledged cost of incontinence and concern about the economic burden of disease [23-25], few studies have examined the economics of surgical procedures for stress urinary incontinence. Papers have examined the costs of TOT versus Burch colposuspension [52,53], and TOT versus other surgical procedures [54]. TOT was found to be more cost-effective than other surgical procedures using RCT data up to 6 months [52], and using economic modeling up to 5 years [54] and 10 years [53].

A single study, using retrospective chart data and a Markov modeling, evaluated the cost of TOT undertaken as an inpatient procedure versus TVT as day surgery procedure, finding the day surgery more cost effective than inpatient surgery [55], but not commenting on the cost effectiveness of TOT versus TVT.

Our own economic evaluation using the data following women to 12 months in our TOT-TVT trial [45], suggested that TOT is a cost effective alternative to TVT in the treatment of SUI. However, these results must be confirmed using longer term follow-up.

#### Summary

The evidence from published studies provides evidence of safety and effectiveness in favour of TVT as a surgical treatment for SUI compared to more traditional approaches. Unfortunately there is insufficient evidence to compare the long-term outcome of TOT versus TVT as a treatment for SUI. Similarly, insufficient economic evidence is available to evaluate cost effectiveness or cost utility of TOT versus TVT. Our proposed study will address these issues.

## Methods/Design

### Trial design

The research is an extension to a trial previously conducted [16]. The new study further evaluates the safety, effectiveness and cost utility of the two devices, TOT and TVT. The study involves follow up of all participants from the initial trial, and includes physical examination and quality of life measurement. In addition, a health economic study is being undertaken. Ethics approval was provided by the University of Calgary Conjoint Health Research Ethics Board, Ethics ID 18421.

### Trial centres

Patients were initially recruited from three Calgary centres, with all follow-up carried out at the Calgary Pelvic Floor Disorders Clinic. All 5 year follow-up visits will similarly be conducted in the Pelvic Floor Disorders Clinic.

### Inclusion/exclusion criteria

All women included in the initial trial will be eligible for inclusion in the 5 year follow-up study.

#### Inclusion criteria

Women were included if they:

• had type II stress incontinence, defined as leaking with increased abdominal pressure [56,57].

• were eligible for both types of surgery.

#### Exclusion criteria

Women were excluded if at the time of the index surgery they:

• had vaginal prolapse requiring surgical repair at the same time as the index surgery

• had previously had incontinence surgery

• had overactive bladder or incontinence caused only by bladder overflow

• intended to have further children

• had Alzheimer's or Parkinson's disease, progressive neurological disease such as multiple sclerosis, or were immunocompromised

• were unable to understand English.

### Trial interventions

Women in the trial received the following interventions [16]:

#### Surgical care

All seven surgeons who participated in the trial were trained to undertake both the TOT and TVT procedures, and had carried out a minimum of 5 of each procedure as lead surgeon after training. At the time of the initial study, the TVT procedure was the surgical standard of care for stress incontinence in Calgary, and remains so to date.

Women in both groups had their surgery performed according to the usual practice of the operating surgeon. Anaesthesia was either general or local, depending on the clinical state and choice of the patient, and according to the usual clinical practice of the anaesthesiologist. Where possible, the operations were planned as day procedures.

#### TOT Group

The TOT procedure was carried out according to the device manufacturer's recommendations.

#### TVT Group

The TVT procedure was carried out according to the device manufacturer's recommendations.

#### Post-operative care for all patients

As usual in Calgary for day case patients, trial participants in the trial were cared for in their homes, unless a hospital stay was required for clinical or administrative reasons.

### Practical arrangements for allocating participants to trial groups

Consenting patients were randomly allocated to receive either a TOT procedure or a TVT procedure. The randomization list was generated by the study statistician (using ralloc procedure in Stata (StataCorp LP, Texas, USA)) using permuted block randomization with block sizes varying from 2 to 8, and stratified by surgeon. Randomization was carried out a few days before surgery, to ensure that the appropriate surgical device was available in the operating room.

### Methods for protecting against sources of bias

The initial trial was undertaken as a pragmatic trial [58]. Neither surgical team nor patient knew the next treatment allocation. Outcome measurement was not carried out blindly, but was conducted by a research nurse who was independent of clinical care. The vaginal examinations to palpate for tape erosion and pain were carried out by a clinician (MR) who had recruited 24 of the study patients. Although not informed of the patient's group of allocation at the time of vaginal examination, it became apparent that palpable tapes tended to be associated with TOT, and it was therefore not possible to maintain examiner blinding.

In the 5 year follow-up, similar considerations will apply. The research nurse who carried out the 12 month contacts and follow-up visits, and is therefore already known to study patients, will contact and follow all women at 5 years. The research nurse remains independent of clinical care. For the 5 year follow-up, all clinical examinations will be carried out by a urogynaecology clinical fellow who will be blinded to both group of allocation, and the presence of palpable tape at 12 months.

### Frequency and duration of follow up

The current study will follow-up all women who participated in the trial at 5 years following surgery.

### Primary and secondary outcome measures

#### Primary outcome

Incidence of vaginal erosion or other serious adverse outcome of surgery (requiring additional treatment) over the 5 years following surgery.

#### Secondary outcomes

Objective outcome of SUI surgery at 5 years after index surgery.

Subjective outcome at 5 years following index SUI surgery.

Point prevalence of palpable tape, vaginal erosion or other serious adverse outcomes at 5 years after index SUI surgery.

### Outcome measures be measured at 5 year follow up

All outcomes will be measured at 5 years following index surgery.

#### Primary outcome: incidence of vaginal erosion or other serious adverse outcome

• **Incidence of vaginal erosion **will be determined by chart review and by standardised digital vaginal exam by a blinded examiner at the time of follow-up. The blinded examiner, a urogynaecology fellow, will be trained to undertake the standardized vaginal exam by MR. Any women who had additional treatment for vaginal erosion (any surgical treatment, or conservative treatment with vaginal estrogen), or who is found to have an erosion on digital exam, will be considered to have had a vaginal erosion.

• **Other serious adverse outcomes **will be defined as those occurring during the 5 years following index surgery which are considered to have a suspected "reasonable causal relationship" [59] with the TOT or TVT procedure. The adverse outcome will have resulted in further inpatient or outpatient treatment, an additional surgical or medical intervention, and/or cause persistent disability or incapacity. Possible adverse outcomes will include persistent groin pain and re-operation for SUI.

Women will be asked to recall any problems they believe they experienced as a result of surgery, and hospital charts will be reviewed for any possible adverse outcomes or complications. Details of additional treatment will be sought from the treating physician if treatment was provided elsewhere.

• **An independent adjudication committee **will be established to adjudicate all instances of a primary outcome (vaginal erosion or other serious adverse outcome). The adjudication committee will consist of three urogynaecologists not involved in the trial, from other institutions than the University of Calgary. They will be provided with all clinical details of the adverse outcome, with the cases blinded to group of allocation. The committee will be asked to judge whether each outcome can be defined as a serious adverse outcome and whether it has a "reasonable causal relationship" with the index surgery. Only if the adjudication committee agrees on both counts, will the adverse outcome be considered a primary outcome.

#### Secondary outcomes

##### Objective outcome of SUI surgery at 5 years after index surgery, measured by pad test

Objective evidence of SUI at 5 years following surgery will be obtained using a standardised pad test (as used in our 12 month follow-up [16]), as follows.

• Retrograde filling of bladder with 300 ml sterile water

• Pre-weighed collecting device (pad) is put on and the test period begins

• During a 15 minute period the subject performs the following activities: walk up and down 1 flight of stairs; standing up from sitting, 10 times; coughing vigorously, 10 times; running on the spot for 1 min; bending to pick up small object from floor, 5 times; washing hands in running water for 1 min

• At the end of the test the collecting device is removed and weighed

• If test is representative, subject undertakes uroflow evaluation (check for voiding dysfunction)

Women will be considered "cured" if the pad weight gain is less than 1 g over the test period. This definition of cure was used in our earlier follow-up [16], allowing comparison with that study. This definition has also been used in other incontinence RCTs [34,60].

##### Subjective outcome at 5 years following index SUI surgery

Definitions of subjective outcomes will be those used in our earlier 12 month follow-up [16].

• ***Subjective cure ***is defined as either 'no' experience of stress incontinence, or if urine loss has been 'no problem at all' or a 'small problem':

In the past 7 days, have you lost or leaked urine when you coughed, laughed, sneezed, lifted, exercised, etc?

[ ] No [ ] Yes

**→ If 'yes'**, how much of a problem has this been for you? ***(Mark ONE ONLY****)*

[ ] No problem at all

[ ] A small problem

[ ] A big problem

The seven day recall period captures information on women who are experiencing regular problematic incontinence.

• ***Incontinence-related quality of life ***is measured using the Urogenital Distress Inventory (UDI-6), a 6 item measure of urogenital distress, and Incontinence Impact Questionnaire (IIQ-7), a 7 item measure of incontinence impact [61]. The UDI-6 produces a single index indicating overall symptom distress and the IIQ-7 produces a single summary index of impact (0 no impact/distress to 100 maximum impact/distress). Both measures were developed for use as outcomes in trials of incontinence treatments: both have been independently validated [62-64], and widely used in a variety of trials [65].

• ***Non-disease specific health related quality of life (15-D) ***is measured (as in our earlier study [16]) using the 15D questionnaire, a validated non-disease specific health-related quality of life (HRQoL) instrument, based on the application of multi-attribute utility theory [66]. It consists of 15 dimensions representing attributes of personal health and activities of daily living. A single index score, with limiting values of 0 (= dead) and 1 (= no health problems), will be derived from the results of each questionnaire by applying Finnish population based utility weights [66]. Evidence suggests that utility weights are applicable across geographical locations [67]. Patients completed the 15D questionnaire at baseline, 6 weeks and 12 months, and will be asked to repeat the questionnaire at 5 years. The index scores of the four observations will be combined using the area under the curve method to derive patient-specific QALYs [67-69]. The mean QALY difference between study groups will be adjusted for the difference between baseline utility scores [68].

##### Point prevalence of palpable tape, vaginal erosion at 5 years after index SUI surgery

Point prevalence of palpable tape and vaginal erosion will be measured by standardised digital vaginal exam by a blinded examiner.

### Sample size and justification

We assume a 20% loss to follow-up from the original study, and therefore we estimate the sample size will be approximately 75 in the TOT and 84 in the TVT group. Possible event rates are noted in systematic reviews, although these estimates are limited by the methodological quality and short-term follow-up of the studies included:

**Table 1:** Adverse outcome rates reported in systematic reviews

Adverse Outcome	Reference	Follow-up	TOT	TVT
Vaginal erosion	Long [50]	≤ 12 months	4%	1%
Groin pain	Long [50]	≤ 12 months	6%	2%
Re-operation rate	Novara [48]	up to 16 months	2%	3%

Assuming that our longer follow-up will lead to increased incidence of adverse outcomes, and because of our concern about the high rate of palpable tapes at 12 months, we estimate that the primary outcome (incidence of vaginal erosion or other serious adverse outcome) could be as high as 27% in the TOT group and 12% in the TVT group. If our estimates prove to be correct, our study would have 70% power to detect such a difference (2-sided p = 0.05%) and the resulting p-value would be 0.016 from the comparison of the two proportions using a chi-square test.

If our estimates of effect size prove to be inaccurate, the study will nonetheless provide important clinical and economic information on which to base treatment decisions, because it is the only trial with this length of systematic outcome evaluation.

### Recruitment rate

All women will be contacted initially by mail by the research nurse who did all the patient contact and follow-up evaluations at 12 months. Following the initial mail contact, which will include a description of the study and a consent form, women will be contacted by telephone to schedule a clinic visit. Women will be contacted for the new study two months before their 5 year anniversary, so that follow-up appointments can be scheduled to coincide as closely as possible with the anniversary of patients' index surgeries. Initial surgeries took place over a 21 month period, and therefore the 5 year follow-up visits will take place over a 21 month period, from October 2010 to June 2012.

We believe that we shall achieve good follow-up rates for this study. At 12 months follow-up, 182/199 (91%) study patients participated. We additionally sought consent from women to contact them again at 5 years postoperatively. We obtained consent from 171/199 patients (86%), with no responses from 28 patients (16 from the TOT group, 12 from the TVT group). Among respondents, no patients refused to be contacted at 5 years. We believe that the non-responding patients may have moved away and are in the process of re-tracing them. If we assume that all the women who have not responded are either lost to the study because they have moved away, or else will refuse to join when we do find them, we shall expect to include 86% of patients. If we assume a further 6% drop-out because they have moved away, died, or are unable to take part, that would leave 80% to take part in the study.

The experienced research nurse who is undertaking the study is already known to all study patients, and has established a good rapport with them. Follow-up at 5 years will be carried out at the Calgary Pelvic Floor Disorders Clinic. We believe that these factors will prove important in achieving good follow-up, by reducing the uncertainty involved in joining the follow-up study [15].

We also considered whether there were additional strategies that would help to ensure good subject participation without being coercive [70]. The only appropriate additional strategy we identified is to use a draw for a gift voucher. This strategy has been previously used in research to enhance recruitment [71], and financial incentives are considered appropriate as long as the amount is not sufficient to put subjects under pressure to join [72-74]. We believe that that a draw will be a useful option to encourage subjects to consider joining our study, while not pressurizing them to do so. The Conjoint Health Research Ethics Board approved the use of a ballot for a spa voucher for $100. Four draws will be carried out during the course of the study, one after each group of 50 women.

### Statistical analyses

Analysis will be by intention to treat, whereby women will remain in the group to which they were allocated by randomization, no matter what surgery they received either during the initial surgery or later during the 5 years postoperatively. In our trial, only one patient did not receive the treatment as allocated, a patient whose TOT procedure was converted to a TVT at the same surgery after urethral muscles were torn [16]. In all analyses, this patient has remained in the TOT group to which she was allocated.

The primary analysis will compare the proportion of patients who have a vaginal erosion or other serious adverse outcome of surgery requiring surgical or other treatment at some point during the 5 year follow-up following index surgery, using a chi-square test. Log binomial regression will be used to adjust the results for any observed imbalances on baseline characteristics. Imputation methods will not be used for missing data from absence of consent or dropout. Results will be reported according to the CONSORT Statement extension for pragmatic trials [58]. In regards to the secondary analyses:

• ***Objective urinary incontinence***: the proportion of patients "cured" (leaking < 1 g urine on pad test) at 5 years following index surgery will be compared using a chi-square test.

• ***Subjective urinary incontinence***: the proportion of patients reporting no/little problematic stress incontinence in the past 7 days, at 5 years following surgery will be compared using a chi-square test.

• ***Incontinence-related quality of life***: the mean scores of UDI-6 and IIQ-7 will be compared between the two groups using analysis of covariance adjusting for baseline score.

• ***Prevalence of palpable tape or erosion: ***the proportion of patients with palpable tape or erosion will be compared between groups using a chi-square test. A McNemar chi-square test will be performed to assess whether there is a relationship between having palpable tape or erosion at 12 months, and having of palpable tape or erosion at 5 years following surgery.

### Frequency of analyses

A single statistical analysis is planned, after all of the patients have been followed up, and all data are complete. Similarly a single cost-utility analysis is planned after all trial, resource utilization and cost data are complete. No subgroup analyses are planned.

### Economic evaluation

Replicating our empirical cost-utility analysis [45] in a study which spans 5 years presents two specific challenges. Since the health effect (measured by QALYs) would be based on only 4 observations (baseline, 12 weeks, 1 year and 5 years following surgery) any differences in health outcome between the study arms that occur between years 1 and 5, but are resolved by the end of year 5, will not be reflected in the aggregate QALY calculation. A second challenge relates to cost. Over the course of the 5 year period it is likely that disease-specific costs will represent only a small proportion of total health care costs, which relate to all causes. In this event, a significant difference in disease-specific costs between the study arms may not be reflected at the level of aggregate total costs.

To address these potential challenges we have outlined two analyses: an empirical analysis which focuses on health service utilization that is disease-specific, and a cost utility analysis that is based on decision analytic modeling, which will allow us to measure health effect in more frequent shorter intervals (e.g. annually) during the follow-up period and incorporate only disease-specific costs.

#### Health service utilization

Data will be sought from a variety of sources to ensure that we capture all condition-specific health services utilization related to repeat surgical intervention, as well as surgery and other treatment related to adverse events.

***Patients ***will be asked if they have needed any treatment for SUI or possible adverse effects of surgery in the 5 years following surgery. This approach may be limited because of recall bias, and therefore it will be only one of a number of approaches.

***Alberta Health Services***, the province-wide provider of health services, provided health care utilization data for our previous study, identifying inpatient and ambulatory hospital services. For the 5-year follow-up, we shall seek information in a similar way, using codes for diagnoses and procedures. For diagnoses we shall use ICD-10-CA, an enhanced version of ICD-10 developed by Canadian Institute for Health Information (CIHI) for morbidity classification in Canada [75]. For procedures we shall use the Canadian Classification of Health Interventions (CCI), the Canadian standard for classifying health care procedures [75]. Together these coding classifications will enable us to limit our evaluation to health service use that is related to possible consequence of SUI surgery.

***Alberta Heath and Wellness (AHW)***, the provincial health department, will be asked to provide data relating to the utilization of physician services. The Physician Payments Data File contains diagnostic data (ICD9-CM) which will allow us to identify condition-specific physician services utilization.

***Health services utilization ***will be described for each group. Comparisons between groups will include number of: visits to treat vaginal erosions; visits for treatments related to adverse events; and repeat surgeries for incontinence. Analyses comparing health services utilization between groups will use non-parametric methods.

#### Cost-utility analysis based on decision analytic modeling

We propose to conduct a cost utility analysis from the public payer perspective with the use of a Markov model. We will simulate the movement of a theoretical cohort of SUI patients through a model structure comprised of health states that are defined by key health outcome or health service events in the surgical treatment of SUI.

**Figure 1:**
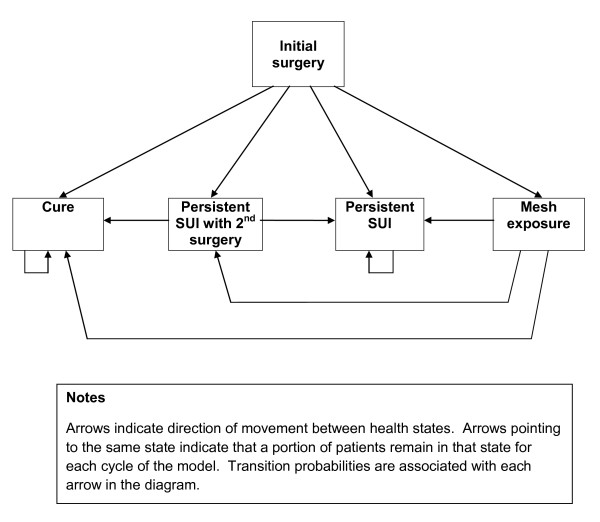
***Illustrative Markov model structure ***[53].

A proportion of the cohort will move to each of the health states once per cycle (i.e. a discrete time period, such as a month or year), in accordance with pre-specified transition probabilities. As an illustration, the structure of a five-state model used in a published evaluation of Burch colposuspension compared with TVT is shown in the Figure 1 [53, figure one].

Quality of life utility weights (with a value between 0 and 1, derived from 15D [66]) and costs are assigned to each health state. Since the transition probabilities sum to 1 for each cycle of the model the expected cost per patient for each cycle is a weighted average of the cost of each of the health states. Summing over the entire time horizon of the model provides the overall expected cost per patient. A similar calculation is performed with utility weights to derive the expected health effect, defined as the average QALY per patient over the study horizon. Expected cost and effect are calculated for each study arm, permitting the calculation of incremental cost and effect. The evaluation will be based on the economic evaluation guidelines set out by the Canadian Agency for Drugs and Technologies in Health (CADTH) [76].

The model shown in the Figure 1 will be the starting point of our model development. Development of the appropriate model structure will be an integral element of this project.

Since only four QALY data observations spread over five years will be available for analysis (our first trial combined with this proposed trial) and our sample size may be too small for some sub-group analyses, we will supplement trial data with data from published sources to estimate transition probabilities and utility weights. For key probabilities, such as the probability of mesh erosion, we will conduct a new meta-analysis as part of this project incorporating all trial data to date.

Disease-specific health state costs will be estimated from administrative data. Utilization data, relating to inpatient and ambulatory hospital services, will be obtained as above. Unit costs related to inpatient and ambulatory care will be obtained from standard cost estimates provided by the CIHI and AHW, respectively. Data relating to the cost of physician services will be obtained from AHW. Costs will be defined with respect to an appropriate reference year. Both costs and QALYs will be discounted at the standard rate of 3% per year.

The uncertainty associated with the estimation of input parameters (transition probabilities, utility weights and costs) will evaluated by a probabilistic sensitivity analysis. This simulation analysis will be used to derive a scatter plot of incremental cost and effect, and a cost-effectiveness acceptability curve. These outputs will be essential in assessing the potential cost-effectiveness of the TOT procedure versus TVT. Additional one-way sensitivity analyses of key parameters and alternative model structures will be undertaken as necessary.

## Discussion

### The need for a trial

This trial is needed now, because TOT and TVT are among the most frequently conducted surgical procedures for SUI in Canada. Because SUI is so common, the impact of selecting an approach that causes more adverse events, or is less effective, will have a significant impact on individual quality of life, and societal and health care costs.

Manufacturers are not required to produce longer term follow-up data to obtain licenses for new SUI devices [7], and therefore this evidence is not available to health authorities, surgeons and patients when they decide on a treatment for SUI. Nor have researchers yet provided longer term evidence to support decision-making.

The results of this follow-up study will inform both clinical choice and set a benchmark against which to judge other surgical trials.

### Use of the results of this trial

The results will produce information about the number of patients who go on to develop vaginal erosions and other serious outcomes. We believe our systematic follow-up of patients at 12 months following random allocation to TOT or TVT surgery, with the observation of palpable tapes in a significant number of patients, is the first such finding, and therefore increasing the duration of follow-up to five years will provide useful information about the longer term safety and effectiveness of the two procedures. These results relating to TOT and TVT procedures will be used by clinicians and patients in informing decisions about surgical treatment of SUI.

The information produced from our cost-utility study can be used by health technology committees (local, regional, provincial or federal) in advising hospitals and clinicians about the use of these procedures, taking into account the cost of the devices and the sequelae of the procedures over a five-year period.

In addition, our study may be able to produce information about any possible link between palpable tape at 12 months after surgery and development of vaginal erosion and other adverse outcomes. Thus our study may be able to provide a better understanding about the development of erosion of surgical tape into the vagina.

## Competing interests

MR, SR and PJ received grant-in-aid research funding from Boston Scientific for the original trial. MR and SR have also received grant-in-aid research funding from Johnson and Johnson and Cook Surgical. MR has acted as a consultant for Cook Surgical. ME, DL declare no competing interests.

## Authors' contributions

All authors contributed equally to the design of the follow-up study, participated in writing the manuscript and have read and approved the final version.

## Authors' information

SR is a health services researcher and Professor in the Departments of Obstetrics and Gynaecology, Family Medicine, Community Health Sciences and Family Medicine, and Director of Research in the Department of Obstetrics and Gynaecology, University of Calgary. MR is a urogynaecologist, Medical Director of the Calgary Pelvic Floor Disorders Clinic and Associate Professor in the Department of Obstetrics and Gynaecology, University of Calgary. DL is a health economist based at the Institute of Health Economics in Edmonton. ME is a biostatistician and Associate Professor in the Departments of Community Health Sciences, Clinical Neurosciences, and Oncology. PJ is a health economist and Professor in the Department of Medicine at the University of Alberta.

## Pre-publication history

The pre-publication history for this paper can be accessed here:

http://www.biomedcentral.com/1472-6874/11/34/prepub
